# Factors Associated With Community Health Worker Performance Differ by Task in a Multi-Tasked Setting in Rural Zimbabwe

**DOI:** 10.9745/GHSP-D-16-00003

**Published:** 2016-06-20

**Authors:** Rukundo A Kambarami, Mduduzi NN Mbuya, David Pelletier, Dadirai Fundira, Naume V Tavengwa, Rebecca J Stoltzfus

**Affiliations:** a Cornell University, Division of Nutritional Sciences, Program in International Nutrition, Ithaca, NY, USA; b Zvitambo Institute for Maternal and Child Health Research, Harare, Zimbabwe

## Abstract

**Background:**

Zimbabwe, like most low-income countries, faces health worker shortages. Community health workers (CHWs) bridge this gap by delivering essential health services and nutrition interventions to communities. However, as workloads increase, CHWs’ ability to provide quality services may be compromised. We studied influences upon CHWs’ performance related to pregnancy surveillance and nutrition and hygiene education in rural Zimbabwe.

**Methods:**

In the context of a cluster-randomized trial conducted in 2 rural districts between November 2012 and March 2015, 342 government-employed CHWs identified and referred pregnant women for early antenatal care and delivered household-level behavior change lessons about infant feeding and hygiene to more than 5,000 women. In 2013, we conducted a survey among 322 of the CHWs to assess the association between demographic and work characteristics and task performance. Exploratory factor analyses of the Likert-type survey questions produced 8 distinct and reliable constructs of job satisfaction and motivation, supervision, peer support, and feedback (Cronbach α range, 0.68 to 0.92). Pregnancy surveillance performance was assessed from pregnancy referrals, and nutrition and hygiene education performance was assessed by taking the average summative score (range, 5 to 30) of lesson delivery observations completed by a nurse supervisor using a 6-item Likert-type checklist. Poisson and multiple linear regressions were used to test associations between CHW demographic and work characteristics and performance.

**Results:**

CHWs who referred more pregnant women were female, unmarried, under 40 years old, from larger households, and of longer tenure. They also perceived work resources to be adequate and received positive feedback from supervisors and the community, but they were less satisfied with remuneration. CHWs with high scores on behavior change lesson delivery were from smaller households, and they received more supportive supervision but less operational supervision. Measures of job satisfaction and motivation were not associated with either task.

**Conclusion:**

Among CHWs responsible for multiple tasks in rural Zimbabwe, factors associated with performance of one task were not the same as those associated with performance of another task. Our methods and findings illustrate ways to examine heterogeneity in CHW performance and to identify organizational factors associated with quality of program delivery.

## Abstract (precis)

Programs should consider specific tasks and how they relate to health worker factors, community support, and the work context. In a setting where community health workers were responsible for multiple tasks, those who referred more pregnant women were female, unmarried, under 40 years old, and from larger households, and they felt they had adequate work resources and positive feedback from supervisors and the community. In contrast, workers with high scores on delivering household behavior change lessons were from smaller households and received more supportive supervision.

## INTRODUCTION

Community health workers (CHWs) are an effective part of the workforce for delivering essential maternal and child health and nutrition services.^1^^,^^2^ Many sub-Saharan African countries such as Zimbabwe face critical health worker shortages, driving the continued expansion of the role of CHWs in these health systems.^3^^,^^4^ CHWs’ scope of practice varies substantially among and within countries; as CHW workload and task complexity increase, concern exists about the quality of services provided by CHWs.^5^^,^^6^

In Zimbabwe, CHWs have been the front line of the national health system since the 1980s.^7^ They provide basic health care treatment and health promotion education on a broad range of topics and report monthly to the head nurse at their nearest primary health care facility. CHWs are selected by their community and are expected to cover a population of about 100 households in their geographical catchment area, working approximately 4 hours per week.^7^

The functionality of the CHW system in Zimbabwe has declined in recent years due to severe economic shocks experienced in the early 2000s.^8^ The social services sector faced funding cuts that contributed to the collapse of health infrastructure including the closure of some government hospitals, drug shortages, brain drain, and the overall deterioration of public health services.^8^ A 2009 report estimated that approximately half of all rural households in Zimbabwe did not have contact with or knowledge of a CHW in their area; furthermore the CHWs lacked basic medicines.^9^ As part of a strategy to strengthen the national health system, the government has been revitalizing the CHW program by increasing recruitment and training of CHWs.^7^^,^^8^

For this health system to work, CHWs need not only to be in place but also to perform at high quality. CHW performance is a complex construct.^10^ Some frameworks describing CHW performance apply a general social ecological perspective, describing the influence of intrapersonal, family, community, and organizational characteristics on CHW performance.^11^^–^^13^ This perspective provides a broad context for situating the individual in the larger environment but provides limited understanding for how the factors interact between levels.^14^

From the field of organizational behavior, goal-setting theory and self-determination theory provide frameworks for understanding the role of CHWs’ extrinsic environments and their intrinsic needs and expectancies at an individual and organizational level.^15^^–^^17^ Goal-setting theory posits that workers identify, commit, and strive to achieve goals and are motivated by clear, challenging goals and appropriate feedback on those goals.^15^ Self-determination theory posits that intrinsic motivation to perform is increased in environments that foster workers’ intrinsic needs (autonomy, competence, and relatedness).^17^

Organizational behavior theories provide frameworks for understanding the role of health workers’ extrinsic environments and intrinsic needs at the individual and organizational level.

Together, these theories link motivation, self-efficacy, knowledge, skills, and goal-setting capacity with performance, and they explain the maintenance of behaviors through a positive feedback, motivation, and effort loop.^15^^,^^17^ However, others also describe the moderating relationship of the nature of the task, especially task complexity, on goal setting and performance.^18^ Thus, different levels of effort are required to perform different tasks. The exertion of these efforts requires the mobilization of different internal (e.g., intrinsic motivation) and external (e.g., work tools) resources. In an enabling context, these efforts are translated into performance.

The nature of tasks, requiring mobilization of different internal and external resources, may affect task performance.

As CHWs’ workloads expand, describing pathways to performance is helpful in understanding the correlates of variation in CHW performance across different tasks and in developing strategies to mitigate poor performance. Therefore, we explored the internal and external resources mobilized by CHWs performing different tasks in the Sanitation Hygiene and Infant Nutrition Efficacy (SHINE) study, a randomized evaluation of several intervention packages delivered by CHWs in rural Zimbabwe. Specifically, the purpose of this article is to investigate (1) CHW demographic and work characteristic factors associated with the performance of 2 different tasks performed by CHWs in the SHINE study, and (2) whether these factors varied by the specific task.

## METHODS

### Study Context

SHINE was a community-based 2x2 factorial cluster-randomized trial conducted in rural Zimbabwe between November 2012 to March 2015.^19^ The trial aimed to determine the independent and combined effects of a water, sanitation, and hygiene (WASH) intervention and improved infant and young child feeding (IYCF) intervention on linear growth and anemia in children born to enrolled pregnant women. Government-recruited CHWs were the backbone of SHINE’s intervention delivery.^20^

As part of SHINE, CHWs conducted early pregnancy identification surveillance every 5 weeks in their catchment areas. For every woman of childbearing age in a CHW’s catchment area, the CHW recorded last menstrual period data on a 5-weekly basis and offered pregnancy tests to women who missed a menstrual period and who assented to the test.^19^ CHWs were also responsible for delivering all behavior change lessons and material inputs as needed (e.g., soap) from the time women were enrolled into SHINE (during early pregnancy) until the new child was 18 months of age. Monthly lesson delivery was aligned with specific gestational age or infant age, to increase relevance for the women and facilitate uptake and modification of key maternal behaviors. CHWs delivered intervention arm-specific messages and standard-of-care messages (that cover Ministry of Health and Child Care [MoHCC] content) to all participants.^19^

All SHINE CHWs received a standard 5-month MoHCC training program. The program consisted of 8 weeks each of in-classroom and field-based training and concluded with a 4-week classroom session following the field internship.^7^ Training covered topics in maternal and neonatal care, HIV/AIDS, tuberculosis, child health and nutrition, non-communicable diseases, WASH, communication, and adult education methods.^7^ Short refresher trainings were mandatory and conducted twice annually if funding was available.^7^ SHINE CHWs were trained for an additional 20–35 days on content specific to the SHINE trial^20^ and on work scheduling and planning to help CHWs integrate SHINE activities into their normal work routines. Experienced MoHCC trainers conducted all trainings, and SHINE staff provided support for the additional SHINE-specific training. All CHWs received a standard monthly MoHCC allowance of US$14, distributed every quarter, along with a SHINE food basket valued at US$42 in token of the additional time CHWs spent on SHINE activities.^19^

Thirty-two SHINE nurse supervisors provided constructive feedback and supervision support for the 342 CHWs. Half of the supervisors were men, half were married, and they ranged in age from 23–48 years. Supervisors met once a month with CHWs for group meetings (approximately 11 CHWs per supervisor) to discuss concerns, troubleshoot, and review expectations. Individual review meetings with CHWs were held in the field approximately once a month, and supervisors evaluated performance of specific tasks and provided additional support.

### Survey Participants and Instrument

A survey was conducted in 2013 among all 342 CHWs living in Chirumanzu and Shurugwi districts participating in the SHINE trial. Two CHWs declined and 18 did not attend the meetings where the survey was administered.

The questionnaire was adapted from a survey used in a previous study^21^ and was modified, translated, and pretested by the study team to fit the Zimbabwean context. The questionnaire included questions about sociodemographic characteristics, motivation, supervisory support, peer support, community and organizational feedback mechanisms, and standard health curriculum knowledge covered in MoHCC training. Questions varied in format, but most used a 5-point Likert response scale ranging from strongly agree to strongly disagree.^22^

The questionnaire was administered to CHWs by 5 trained Zimbabwean enumerators (fluent in English and the local language Shona), and data were captured electronically using netbook computers.^19^^,^^20^ Data were collected over 20 days in April 2013 while CHWs attended SHINE trainings. On average, 16 questionnaires were administered to CHWs each day, with each interview lasting 45–60 minutes.

### Ethical Approval

Written informed consent was obtained from CHWs in their preferred language before administering the questionnaire. Ethical approval for the study was provided by the Medical Research Council of Zimbabwe and the Johns Hopkins Bloomberg School of Public Health Institutional Review Board.

### Performance Outcomes

We selected pregnancy referral rate and behavior change lesson delivery as important and contrasting performance outcomes and because of their relevance to other maternal and child health interventions. The major differences between the 2 outcomes were the novelty and prestige of the task (higher for pregnancy referrals), CHW and community cultural acceptability for the task (higher for lesson delivery), and time burden for the task (higher for lesson delivery) (Supplementary Table 1).

We focused on health workers’ performance related to pregnancy referral rates and behavior change lesson delivery.

#### Assessment of Pregnancy Referrals

Pregnancy referral data were obtained from the SHINE database. CHWs regularly visited all women of reproductive age in their area and identified new pregnancies through a 2-stage process of asking about the last menstrual period and confirming pregnancies with a dipstick urinary hCG test (Pregnancy Midstream Tests, Kurkel Enterprises, LLC). Pregnant women were referred to clinics for antenatal care, including HIV testing and care. Pregnancy referral information was also sent to a SHINE supervisor who arranged for a second, confirmatory urine test administered by a research nurse. Confirmed (i.e., SHINE-validated) CHW pregnancy referrals were entered daily into the SHINE database. Total referrals in 2013 were summed for each CHW, and data on the median number of women 15–49 years per CHW catchment area were extracted from CHW registers. Referrals per women of reproductive age were calculated as an annual rate. Pregnancy referral data were available for 319 CHWs.

#### Assessment of Behavior Change Lesson Delivery

Nurse supervisors assessed the quality of behavior change lesson delivery during a supervisory visit planned to occur during one of the first times that a specific lesson was being delivered. The supervisor observed the CHW delivering a lesson and completed a checklist of 6 statements about adherence to quality lesson delivery:

1. Reviewed last session with mother
2. Asked mother questions about her recall, knowledge, and current practices
3. Delivered lesson in a relaxed manner
4. Allowed mother to ask questions
5. Responded to mother’s questions correctly and appropriately
6. Reviewed current lesson information at the end of the session

The supervisors rated each checklist statement on a 5-point Likert scale,^22^ and a summative score (range, 5–30 points) was later calculated by the researchers (Cronbach α = 0.83). Supervisors provided feedback to the CHW after each observation. In the SHINE Trial, which aimed to evaluate the effects of 2 different CHW content packages in a 2x2 factorial design (4 intervention arms), the CHWs delivered 15 different behavior change lessons.^19^ However, the instrument assessed issues of quality that were relevant to every lesson, regardless of content. For each CHW, the average lesson score was calculated from all lessons observed (median, 6 lessons; interquartile range, 3–10) between January 2013 and August 2014. Lesson delivery score data were available for 289 CHWs; all were included in this analysis.

### Statistical Analyses

Descriptive statistics were performed on CHW demographic data. Exploratory factor analysis was used to reduce data from the questionnaire. From each section, factors were retained after principal axis factor extraction, scree tests, and promax rotation.^23^ Items with factor-loadings above 0.30 were included in factors and any items that cross-loaded were included in the factor where they had the higher factor-loading and conceptual relevance. Reliability analyses were performed on factors, and items that decreased reliability were omitted from the final factors. For each factor, the total score was calculated as the sum of scores for the items in the factor. Factors were standardized as *z* scores to account for the different number of items in each factor. Across factor variables, the proportion of CHWs missing data was 15% (n = 49). Missing data from factors were imputed using multiple imputation by chained equations with 10 iterations and all model covariates with non-missing data specified.^24^

Multiple linear regression was used to investigate associations between CHW lesson delivery score and demographic and factor variables. Models were adjusted to account for the different number of lesson observations per CHW. Poisson regression was used to assess associations with the number (count) of pregnant woman referred over the 1-year period. To account for the different number of women of childbearing age in each CHW’s catchment area, a natural log of median number of women of childbearing age was included in the model as an offset. Linearity of continuous independent variables was examined for each outcome by scatter plot smoothing.^25^ For pregnancy referral rate, the job satisfaction and motivation factor was modeled as a categorical variable. All other variables were modeled as linear variables for both regression models.

All demographic variables and standardized factors were entered into a backwards-stepwise regression analysis. Level of significance was at the *P* = .05 level. Models were adjusted for SHINE trial randomization variables and demographic variables of interest from CHW performance literature.^12^ To assess differences among the variables associated with performance between the 2 tasks, we fit a multilevel linear model.

Multilevel modeling was used to account for the non-independence of performance scores for the same CHWs nested within nurse supervisors. CHW and nurse supervisor were identified as random effects, and CHW demographic and work characteristics were modeled as fixed effects. To compare predictors between the different tasks, first we transformed the pregnancy referrals data using a square root transformation because it followed a Poisson distribution. Then we standardized as *z* scores the lesson delivery score and the transformed pregnancy referrals data. We created a dummy variable for the task and tested the interaction term between the dummy variable and each demographic and work characteristic variable. This interaction model was adjusted for SHINE trial randomization variables, the number of lesson observations per CHW, and the median number of women of childbearing age in a CHW’s catchment area. All data were analyzed using STATA 12.0.

## RESULTS

### CHW Demographic and Work Context Factors

Nearly three-quarters (73.6%) of the 322 CHWs were women, most were married (76.7%) and of middle age (mean, 45.0 years ± 8.8 years), and 83.3% had some secondary school experience or higher (Table 1). The median duration of job tenure was 3.2 years, and for 75.5% of CHWs, this was their first experience with a health education job.

**TABLE 1:** Background Characteristics of Community Health Workers, Shurugwi and Chirumanzu Districts, Zimbabwe, 2013 (N = 322)

Characteristic	Value
Age, mean ± SD, years	45.0 ± 8.8
<40, No. (%)	87 (27.0)
40–50, No. (%)	153 (47.5)
>50, No. (%)	82 (25.5)
Gender, No. (%)	
Female	237 (73.6)
Marital status, No. (%)	
Currently married	247 (76.7)
Other	75 (23.3)
Household size, mean ± SD	4.82 ± 2.50
Educational level, No. (%)	
Primary (7 years)	54 (16.8)
Some secondary (8–10 years)	101 (31.4)
“O” Level^a^ or higher (11+ years)	167 (51.9)
Tenure as CHW, median (IQR), years	3.2 (2.2, 11.1)
First experience with health education job, No (%)	243 (75.5)

Abbreviations: IQR, interquartile range; SD, standard deviation; CHW, community health worker.

^a^ Ordinary, or “O”, Level certificate examination is a terminal examination taken after 4 years of secondary education.

Eight factors emerged from factor analyses of work characteristic questions (Table 2):

- Job satisfaction and motivation
- Satisfaction with remuneration
- Perceived peer support
- Perceived supportive supervision
- Perceived operational supervision
- Perceived adequacy of resources for work
- Perceived negative feedback (from supervisors, community, and peers)
- Perceived positive feedback (from supervisors, community, and peers)

**TABLE 2:** Description of 8 Work Characteristic Factors Emerging From Factor Analysis of CHW Survey Responses, Shurugwi and Chirumanzu Districts, Zimbabwe, 2013

Factor	Number of Survey Items	Description of Factor
Perceived job satisfaction and intrinsic motivation	12	Feels personally motivated and happy with work and making a positive impact; feels appreciated by community, health workers, and organization for work
Satisfaction with remuneration	3	Satisfied with remuneration for the work
Perceived peer support	5	Receives advice and support from other CHWs
Perceived supportive supervision	12	Feels valued, motivated, guided, and heard, and feels supervisor is accessible
Perceived operational supervision	10	Feels informed and consulted about work activities; received communication to improve work; feels needs are represented
Perceived adequacy of resources for work	5	Frequency of shortage of transportation and work tools
Perceived negative performance feedback	5	Community, other CHWs, and supervisor attitude indicate poor performance
Perceived positive performance feedback	6	Supervisor and community inform CHW of good performance, positive changes in community; increased job confidence

Abbreviation: CHW, community health worker.

Reliability analyses showed adequate internal consistency for all scales (Cronbach α≥0.70) except for perceived positive performance feedback (α = 0.68) (Table 3). Mean scores on 5 of the 8 factors were high, at or above 75%. Negative feedback and satisfaction with remuneration mean factor scores were moderate, at 70% (mean score of 17.6 out of a maximum score of 25) and 66% (mean score of 9.9 out of maximum of 15), respectively. Adequacy of resources for work had the lowest mean score at 63%. CHWs’ knowledge scores were moderate (mean of 17.6 out of 24 questions), and less than 1% of CHWs scored below 12 out of 24 questions (Table 3).

**TABLE 3:** Reliability of Scales Assessing CHW Work Characteristic Survey Questions, Shurugwi and Chirumanzu Districts, Zimbabwe, 2013

Work Characteristic (Range of Scale) (No. of Respondents)	Cronbach alpha	Range in Data	Mean ± SD
Health curriculum knowledge (1–24) (N = 322)	–a	11–22	17.6±2.0
Job satisfaction and motivation (12–60) (N = 311)	0.85	39–60	53.0±4.7
Satisfaction with remuneration (3–15) (N = 318)	0.92	3–15	9.9±3.4
Perceived peer support (5–25) (N = 316)	0.75	5–25	18.7±4.4
Perceived supportive supervision (12–60) (N = 314)	0.90	31–60	51.7±5.7
Perceived operational supervision (10–50) (N = 310)	0.77	22–50	42.6±5.7
Perceived negative feedback (5–25) (N = 307)	0.72	7–25	17.6±3.6
Perceived positive feedback (6–30) (N = 315)	0.68	16–30	25.3±2.5
Perceived adequacy of resources for work (5–25) (N = 312)	0.70	5–25	15.7±4.3

Abbreviations: CHW, community health worker; SD, standard deviation.

a Knowledge items were based on training materials and do not reflect a singular “knowledge” construct; therefore, we do not report a Cronbach alpha.

### Pregnancy Referral Rate

Several CHW characteristics were significantly associated with more pregnancy referrals in the imputed model (Table 4): female gender (incidence rate ratio [IRR] for referring an additional woman = 1.13; *P*<.05), larger household size (IRR = 1.01; *P*<.05; range, 0–16 people), and longer job tenure (IRR = 1.01; *P*<.01; range, 1–30 years). Middle-aged (IRR = 0.89; *P*<.05) and older CHWs (IRR = 0.81; *P*<.01), as well as those who were married (IRR = 0.88; *P*<.05), made fewer referrals.

**TABLE 4:** Poisson Regression Models to Predict Factors Associated With the Pregnancy Referral Rate (in 1 Year) of CHWs, Shurugwi and Chirumanzu Districts, Zimbabwe, 2013

CHW Variables	Pregnancy Referral Rate			
	Complete-Case Model (N = 299)		Imputed Model (N = 319)	
	IRR (95% CI)	*P* Value	IRR (95% CI)	*P* Value
Age, years (reference: <40)				
40–49	0.89 (0.80, 0.99)	.04*	0.89 (0.80, 0.98)	.02*
≥50	0.81 (0.70, 0.93)	.002**	0.81 (0.71, 0.93)	.002**
Gender (reference: male)	1.14 (1.02, 1.26)	.02*	1.13 (1.02, 1.25)	.02*
Marital status (reference: not married)	0.89 (0.80, 0.98)	.02*	0.88 (0.80, 0.97)	.01*
Household size	1.02 (1.00, 1.03)	.07	1.01 (1.00, 1.03)	.047*
Tenure, years	1.01 (1.00, 1.02)	.003**	1.01 (1.00, 1.02)	.003**
Satisfaction with remuneration	0.92 (0.87, 0.96)	<.001***	0.92 (0.88, 0.96)	<.001***
Perceived adequacy of resources for work	1.06 (1.02, 1.11)	.008**	1.06 (1.02, 1.11)	.006**
Perceived positive feedback	1.06 (1.01, 1.11)	.01*	1.06 (1.01, 1.10)	.01*

Abbreviations: CI, confidence interval; IRR, incidence rate ratio; CHW, community health worker.

Models adjusted for CHWs’ education, knowledge, study arm, cluster ID, ward number, and median number of women of childbearing age per CHW catchment area.

Significant at

* *P*<.05;

** *P*<.01;

*** *P*<.001.

From CHW perceptions of their work characteristics, positive feedback (IRR = 1.06; *P*<.05) and adequacy of work resources (IRR = 1.06; *P*<.05) were the only factors significantly associated with more referrals. Interestingly, CHWs who were more satisfied with remuneration made fewer referrals (IRR = 0.92; *P*<.001).

### Behavior Change Lesson Delivery Score

Multiple regression analysis showed 2 variables were significantly associated with lesson delivery score (Table 5): supportive supervision was associated with higher scores (B, 0.41; *P*<.05), but operational supervision was associated with lower scores (B, -0.43; *P*<.05).

Supportive supervision was significantly associated with higher behavior change lesson scores, while operational supervision was significantly associated with lower scores.

**TABLE 5:** Multiple Linear Regression Models to Predict Factors Associated With Behavior Change Lesson Delivery Scores^a^ of CHWs, Shurugwi and Chirumanzu Districts, Zimbabwe, 2013

CHW Variables	Lesson Delivery Score			
	Complete-Case Model (N = 274)		Imputed Model (N = 289)^a^	
	Beta (SE)	*P* Value	Beta (SE)	*P* Value
Household size	-0.14 (0.08)	.05	-0.12 (0.07)	.08
Tenure, years	-0.05 (0.03)	.13	-0.05 (0.04)	.11
Perceived supportive supervision	0.43 (0.21)	.04*	0.41 (0.20)	.04*
Perceived operational supervision	-0.45 (0.21)	.03*	-0.43 (0.20)	.04*

Abbreviations: CHW, community health worker; SE, standard error.

a Based on supervisor’s summative score to six 5-point Likert response statements: reviewed last session with mother; asked mother questions about her recall, knowledge, and current practices; delivered lesson in a relaxed manner; allowed mother to ask questions; responded to mother’s questions correctly and appropriately; and reviewed current lesson information at the end of the session.

Models adjusted for age, gender, marital status, education, knowledge, study arm, cluster ID, ward number, and number of observations per CHW.

Significant at

* *P*<.05

** *P*<.01;

*** *P*<.001.

### Interaction Model

The type of task significantly modified the relationship between work performance and some CHW demographic and work characteristic variables (Supplementary Table 2). Significant interactions were observed for CHW gender, household size, job tenure, work resources, and operational supervision. Higher task performance on pregnancy referrals (more referrals) was associated with female gender (*P*<.05), larger household size (*P*<.05), more job tenure (*P*<.01), more operational supervision (*P*<.05), and the availability of resources for work (*P*<.10) (Figure). However, the same variables were associated with *lower* task performance on lesson scores (lower scores) (Figure). The magnitudes of effect were weak for household size (*z* score< 0.10), tenure (*z* score<0.10), and adequate work resources (*z* score<0.14), and moderate for perceived operational supervision (*z* score<0.15) and gender (*z* score<0.32).

The same variables that were associated with higher task performance on pregnancy referrals were associated with lower task performance on behavior change lesson scores.

**FIGURE:**
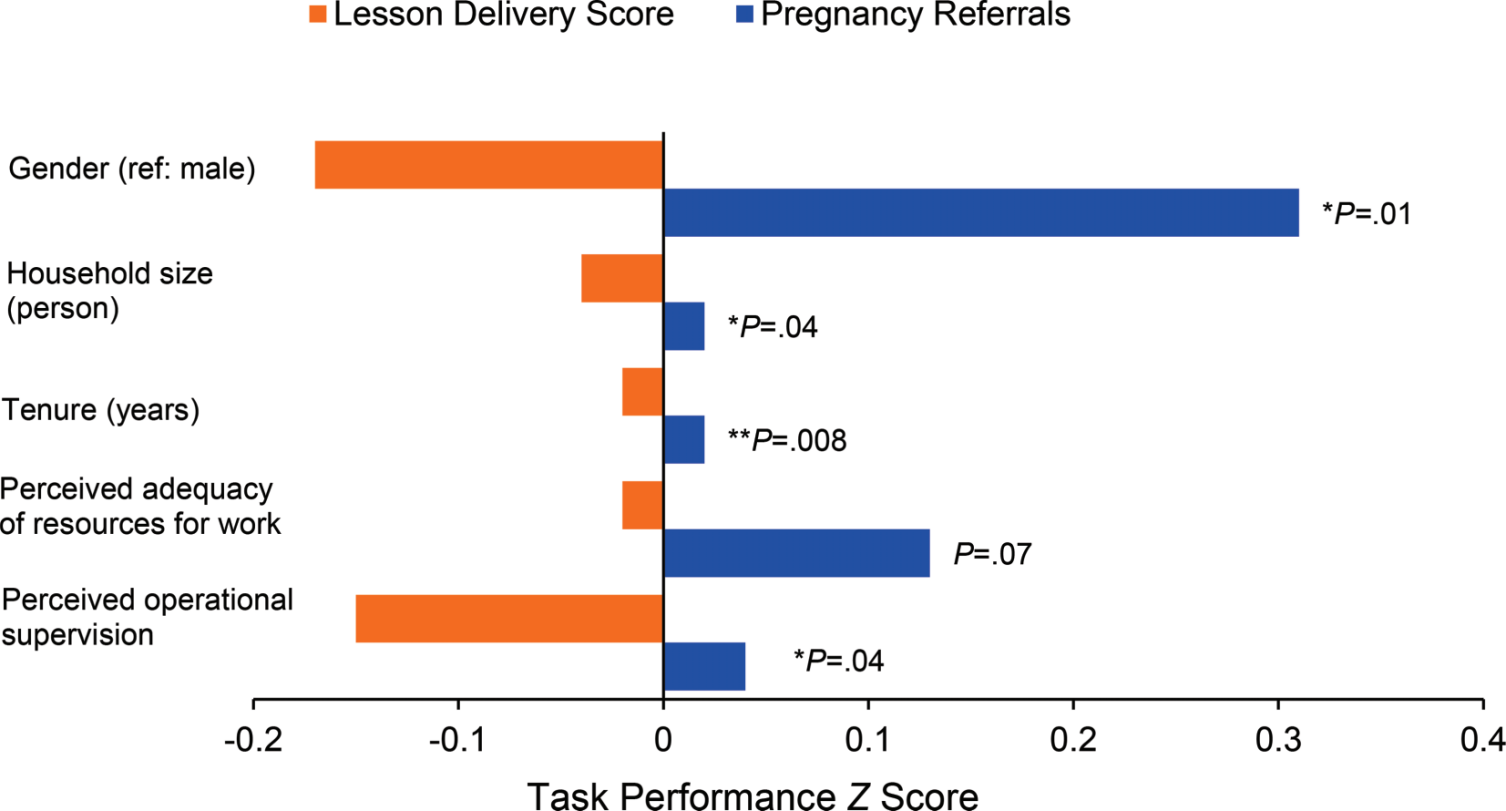
Significant Interaction Terms of Task and Demographic and Work Characteristics From the Interaction Model, Shurugwi and Chirumanzu Districts, Zimbabwe, 2013

Significant at *P<.05; **P<.01; ***P<.001.

## DISCUSSION

In this context of well-trained and supported CHWs in rural Zimbabwe, different individual and organizational factors were associated with the performance of different tasks. Performance related to pregnancy referrals was associated with several CHW demographic characteristics (age, gender, marital status, and household size) and several work characteristics (financial incentives, feedback, resources, and job tenure) while performance on behavior change lesson delivery was associated with only 1 demographic characteristic (household size) along with supervision factors. The factors that proved significant for these Zimbabwean CHWs have also been highlighted by other authors,^5^^,^^12^ but there have been no studies considering how factors differ by the type of task. Our findings suggest the need for programs to tailor approaches to improve CHW performance by carefully considering the different factors associated with various tasks that CHWs perform. Moreover, for researchers it reinforces the need to explore how various factors lead to improved performance for different tasks.

### Factors Associated With Pregnancy Referrals

Female CHWs made more pregnancy referrals than male CHWs. In many cultures, early pregnancy detection is a culturally sensitive topic,^26^ and it may be easier for women to disclose their pregnancy to female CHWs. Other studies have shown gender to positively influence performance on maternal and child health activities,^27^^–^^29^ consistent with our findings. The fact that the gender and task interaction was significant further illustrates that for different types of tasks, women perform the task differently than men.

Female community health workers made more pregnancy referrals than male workers.

For age and marital status, there is no consensus in the literature regarding associations with health worker performance, and it seems plausible that the influence of these factors varies by task and social context.^12^ In this Zimbabwean context, younger (<40 years old) and unmarried CHWs performed more pregnancy referrals. CHWs’ younger age may increase their accessibility to women of childbearing age and their physical capacity to canvass villages. Unmarried CHWs may have less time demands and be able to conduct more pregnancy referrals. Variability in task time burden and task complexity can influence which characteristics are associated with better performance.

CHWs with larger households (adjusted for age) made more pregnancy referrals. Larger households could be a proxy for family support,^30^^,^^31^ and a few studies suggest CHWs with fewer household duties and family support may be more productive.^30^^,^^31^ There was also a significant interaction between pregnancy referrals and job tenure (adjusted for age). Limited and mixed evidence suggests more job experience may improve client satisfaction and CHW use of tools.^32^^,^^33^ The novelty and social prestige associated with the pregnancy identification task may increase community and family support and the sharing of household duties,^30^^,^^31^^,^^34^ giving CHWs time to work and motivating CHWs to perform.

Despite numerous studies reporting that financial incentives are associated with motivation and improved performance,^12^^,^^35^^–^^37^ CHWs who were more satisfied with their remuneration made fewer pregnancy referrals. SHINE CHW allowances were higher than the ordinary government allowance and were also independent of the workload and performance. Thus, low performers, satisfied with their allowances, may have found no added incentive to performing the new task. A similar finding among CHWs delivering behavior change education in rural Haiti led those authors to posit that the CHWs perceived little benefit to increasing performance in face of the difficult terrain, an observation they termed “disgruntled stars and happy slackers.”^38^ Similarly rugged working conditions exist in rural Zimbabwe that could explain our results. However, it is important to note that this observation applied to the new task and it could also be a function of workload. This has implications for increasing CHW workload, particularly in difficult-to-reach, resource-limited settings. CHWs work under challenging conditions and livelihood constraints in many parts of the world. In fact, SHINE and the MoHCC initiated a performance-based incentive scheme after this study was completed, and these data are currently being evaluated.

Adequate work resources were associated with more pregnancy referrals, a finding consistent with several studies.^12^ Pregnancy identification required CHWs to have pregnancy test kits to make referrals. The nature of the task influenced how CHWs perform on tasks, as the significant interaction of work resources and task type confirmed. When tools are essential to the performance of a task, the lack of those tools certainly constrains performance.

Positive feedback from supervisors and the community was associated with improved performance for pregnancy referrals. Our finding is consistent with prior studies linking supervision and recognition with improved motivation and performance.^31^^,^^39^^–^^41^ The pregnancy identification task allowed CHWs and women to find out women’s pregnancy status immediately, and CHWs likely received immediate feedback from the women. Additionally, the supervisor’s skill providing constructive positive feedback may have motivated CHWs to perform this new activity. Supervision is important for all tasks; but for different tasks, specific aspects of supervision are vital to improved performance, reinforcing the importance of different supervision approaches.

### Factors Associated With Behavior Change Lesson Delivery

Fewer demographic and work characteristic items predicted lesson delivery scores than pregnancy referral completions. CHWs from larger households (adjusted for age) had lower scores—a finding exactly opposite from those for pregnancies referrals. Lesson delivery is a routine activity, takes approximately an hour to complete, and lacks the social prestige of newer, faster-to-complete tasks, which may reduce family support and household duty redistribution, resulting in lower lesson scores for CHWs with large families. In addition, CHW fatigue is also likely to decrease motivation to perform, particularly in the absence of family and community support.^34^

We assessed perceptions of two types of supervision, supportive and operational, and they were oppositely associated with lesson delivery. Supportive supervision, characterized by treating CHWs as peers and supporting their work needs, was positively associated with lesson delivery. Operational supervision, characterized by frequent interactions and consultations for work, was inversely associated with lesson delivery. Because of the routine nature of lesson delivery, CHWs may have perceived operational support as disruptive and a hindrance to performance, as indicated by the interaction of operational supervision and task type. It is also possible that supervisors intervened with weaker CHWs by providing more operational support than supportive supervision (reverse causality in our analysis). How CHWs perceive different supervision approaches is just as important as the supervision package itself. Further research is required to unpack supervision and understand what types of supervision approaches are appropriate for different tasks to promote improved CHW performance.^12^^,^^42^

Interestingly, the item we termed “job satisfaction and motivation” was not associated with either pregnancy referrals or behavior change lesson delivery. This factor contained items representing recognition, satisfaction, and intrinsic motivation. Although these items loaded as one factor and had an adequate Cronbach’s alpha, it may have been too broad. A better scale separating out these constructs might provide more clarity for the relationships between the tasks and constructs.

### Theoretical and Practical Implications

Our work suggests that pathways to health worker performance for different tasks may vary, which has implications for contexts where CHW responsibilities are expanding. Importantly our findings show there is no single strategy that can be used to improve performance. There is need for broader conceptual thinking about CHW performance, the specific tasks they perform, and the environments in which they perform these tasks. Theories of motivation and performance are drawn from settings where there are clear roles and organizational structures and where workers are paid for their services. However, in low-income settings where CHWs are often unpaid, have less clearly defined roles, and have dual responsibility to their community and the health system, these theories may require adaption to fit the context. Further research examining CHW motivation and performance that acknowledge specific activities or groups of activities can begin to take cognizance of the complexity of CHW performance and help to develop relevant theoretical frameworks that can guide the design and strengthening of programs.

There is no single strategy that can be used to improve task performance.

Where CHWs have multiple concurrent responsibilities, selecting or recruiting CHWs based on the type and number of tasks within their purview is a substantial challenge. For practical, logistical, and political reasons, it might be more feasible to use such information for targeting supportive supervision and mentoring for performance improvement.

From a CHW training perspective, practices that include targeting CHWs based on gender or tenure and offering additional or special trainings for some groups of CHWs based on specific tasks may facilitate improved task performance. For CHW supervision, training CHW supervisors on supervision approaches that are less didactic and more interactive—where CHWs feel they are heard and guided—can foster improved performance. In addition, monitoring supervisor feedback and interactions with CHWs will also help ensure supervision is constructive. For those CHWs that require extra supervision, it is even more important to tailor the supervision approach to meet supervisory goals without negatively affecting CHW performance.

CHW programs in Zimbabwe and elsewhere can consider supporting CHWs based on meta-characteristics that include the structural and social aspects of the work environment. A meta-characteristic profiling/targeting of CHWs would be one that considers the requirements for the job tasks, for example, time to conduct the activities, community value for the health activities, cultural norms about the health activities, the size of population to be covered, and family support for the CHW’s work. Using this information, the program can navigate appropriate CHW strengthening to promote improved task performance. National CHW surveys can help programs answer the question of what services CHWs actually provide. With this knowledge, programs can then examine various drivers for task-specific performance.

Overall, improving CHW performance in multi-task environments requires building a facilitative environment. This means using community-wide interventions that integrate CHW demographic factors, the community, and the work context. This package of interventions can address CHW training, supervision, remuneration, household-level dynamics, and community sensitivities for different tasks to create a conducive environment for CHWs to perform tasks.

Improving performance of health workers responsible for multiple tasks requires building a facilitative environment.

### Limitations

Our findings should be interpreted taking into account certain limitations of the study. First, the cross-sectional design does not allow for causal inference nor permit inference about the direction of associations and for how different factors change over time. A longitudinal cohort study would be the ideal design to examine directionality and how the relationships change over time.

Second, the behavior change lesson delivery checklist was a subjective measure of performance and more vulnerable to random error and rater bias than pregnancy referral performance—an objective measure.^43^^,^^44^ We attempted to minimize any rater bias that could either inflate or deflate estimates by training the nurse supervisors on a standardized procedure to complete the lesson delivery checklist.^20^ While objective measures are often more reliable, they reflect the results of behaviors, whereas subjective measures reflect the actual behaviors,^45^ which was key for assessing the *quality* of lesson delivery. Using lesson scores from multiple observers unfamiliar with the CHW could help increase precision and reduce potential bias; however, this was not possible in our study.

Third, to minimize potential social desirability bias from the CHW interviews, the data collectors were trained in interviewer neutrality and followed a standard operating procedure for administering the interview.^20^ Finally, these results take place within an efficacy study with adequate resources and supported by national and local government entities in the 2 districts. The CHWs received an extensive amount of support, with an emphasis on a relatively small number of tasks, which limits the external validity of our results in other countries and contexts, such as government programs, that have fewer resources. Nevertheless, our results highlight that the factors shaping performance vary for different types of task.

## CONCLUSION

CHW services are important in ensuring the delivery of primary health care services in many low-income settings. We studied factors associated with CHW performance on contrasting jobs tasks and found that the factors associated with performance differed by task. This suggests that in multi-task environments, what works to improve performance for some tasks may not work for other tasks. As such, CHW programs should consider creating facilitative work environments that include developing familial and community support for CHW tasks and addressing CHW needs, including appropriate remuneration. Also, focusing on understanding drivers of CHW performance in multi-task settings can help to prevent overburdening CHW workloads and to maintain quality CHW performance as countries seek a transition toward universal health coverage.^46^

What works to improve performance for some tasks may not work for other tasks.

## Supplementary Material

Supplementary Table 1

## Notes

**Peer Reviewed**
