# Supplementary material for: Factors Associated With Community Health Worker Performance Differ by Task in a Multi-Tasked Setting in Rural Zimbabwe
Source: Glob Health Sci Pract. 2016 Jun 20;4(2):238–50. doi: 10.9745/GHSP-D-16-00003 (PMC4982248; doi:10.9745/GHSP-D-16-00003)
Supplement: Supplementary Table 1 [file GHSP-D-16-00003_index.html]

Supplement to Factors Associated With Community Health Worker Performance Differ by Task in a Multi-Tasked Setting in Rural Zimbabwe | Global Health: Science and Practice

## GHSP-D-16-00003 Supplementary Table

Kambarami et al. doi: 10.9745/GHSP-D-16-00003

- Supplementary Table 1 - Kambarami et al. doi: 10.9745/GHSP-D-16-00003
- Supplementary Table 2 - Kambarami et al. doi: 10.9745/GHSP-D-16-00003
