# Supplementary material for: Factors Associated With Community Health Worker Performance Differ by Task in a Multi-Tasked Setting in Rural Zimbabwe
Source: Glob Health Sci Pract. 2016 Jun 20;4(2):238–50. doi: 10.9745/GHSP-D-16-00003 (PMC4982248; doi:10.9745/GHSP-D-16-00003)
Supplement: Supplementary Table 1 [file 16-00003-Kambarami-Supplementary-Table-1.pdf]

Kambarami RA, Mbuya MNN, Pelletier D, Fundira D, Tavengwa NV, Stoltzfus RJ. Factors associated with community health worker performance differ by task in a multi-tasked setting in rural Zimbabwe. Glob Health Sci Pract. 2016;4(2). http://dx.doi.org/10.9745/GHSP-D-16-00003

SUPPLEMENTARY TABLE 1. Key Differences Between CHW Tasks

Pregnancy Referrals	Lesson Delivery
New activity in community	Common activity in community
Cultural sensitivity for activity	Culturally acceptable
Visit all homesteads in CHW catchment area every 5 weeks covering seasonally difficult terrain	Visit all SHINE homesteads at least once a month (scheduled visits) covering seasonally difficult terrain
Instruct women how to use and interpret pregnancy tests	Provide demonstrations and use lesson books for visits
Social prestige is likely due to pregnancy test kits and visibility	Fatigue is likely because it is a common activity
Similar procedure for all visits	Different lessons at every visit
Relatively quick visit (~10 minutes)	Longer visits (~60 minutes)
Delivered to women of childbearing age in the household	Delivered to SHINE women and occasionally other household members

Abbreviations: SHINE, Sanitation Hygiene and Infant Nutrition Efficacy; CHW, community health worker.
