# Supplementary material for: Factors Associated With Community Health Worker Performance Differ by Task in a Multi-Tasked Setting in Rural Zimbabwe
Source: Glob Health Sci Pract. 2016 Jun 20;4(2):238–50. doi: 10.9745/GHSP-D-16-00003 (PMC4982248; doi:10.9745/GHSP-D-16-00003)
Supplement: Supplementary Table 1 [file 16-00003-Kambarami-Supplementary-Table-2.pdf]

Kambarami RA, Mbuya MNN, Pelletier D, Fundira D, Tavengwa NV, Stoltzfus RJ. Factors associated with community health worker performance differ by task in a multi-tasked setting in rural Zimbabwe. Glob Health Sci Pract. 2016;4(2). http://dx.doi.org/10.9745/GHSP-D-16-00003

SUPPLEMENTARY TABLE 2. Multilevel Interaction Model Showing CHW Demographic and Work Characteristic Factors Associated With CHW Performance on Pregnancy Referrals and Behavior Change Lesson Delivery Tasks

	Pregnancy Referrals (N=246)		Lesson Delivery Score (N=246)		Interaction Term
**CHW Variables**	**Beta (SE)**	***P* Value**	**Beta (SE)**	***P* Value**	***P* Value**
Age, years (reference: <40)					
40–49	-0.24 (0.14)	.08	0.07 (0.14)	.63	.60
≥ 50	-0.27 (0.19)	.14	-0.14 (0.19)	.45	.42
Gender (reference: male)	0.31 (0.14)	.02	-0.17 (0.14)	.23	.01*
Marital status (reference: not married)	-0.14 (0.14)	.30	0.01 (0.14)	.97	.42
Educational level (reference: primary)					
Some secondary	0.11 (0.11)	.53	0.06 (0.17)	.73	.36
Completed “O” Level[a] or higher	0.01 (0.19)	.98	0.24 (0.19)	.20	.12
Household size	0.02 (0.02)	.30	-0.04 (0.02)	.09	.04*
Tenure, years	0.02 (0.01)	.09	-0.02 (0.01)	.05	.008**
Job satisfaction and motivation	-0.12 (0.08)	.12	-0.07 (0.08)	.34	.66
Satisfaction with remuneration	-0.12 (0.07)	.07	0.02 (0.07)	.74	.11
Perceived peer support	-0.05 (0.06)	.46	-0.04 (0.06)	.57	.90
Perceived supportive supervision	0.02 (0.09)	.82	0.17 (0.09)	.046	.19
Perceived operational supervision	0.04 (0.07)	.53	-0.15 (0.07)	.03	.04*
Perceived adequacy of resources for work	0.13 (0.06)	.03	-0.02 (0.06)	.73	.07§
Perceived negative feedback	0.00 (0.06)	.99	-0.09 (0.06)	.14	.28
Perceived positive feedback	0.15 (0.08)	.06	0.03 (0.08)	.71	.26

Abbreviations: SE, standard error; CHW, community health worker.

Models adjusted for study arm, cluster ID, ward number, and number of observations per CHW.

Interaction terms significant at: §*P*<.10; **P*<.05; ***P*<.01; ****P*<.001.

[a] Ordinary, or “O”, Level certificate examination is a terminal examination taken after 4 years of secondary education.
